# Nuclear Receptor PXR Confers Irradiation Resistance by Promoting DNA Damage Response Through Stabilization of ATF3

**DOI:** 10.3389/fonc.2022.837980

**Published:** 2022-03-16

**Authors:** Xiaxia Niu, Hongmei Cui, Xinsheng Gu, Ting Wu, Min Sun, Changlong Zhou, Mei Ma

**Affiliations:** ^1^ Institute of Toxicology, School of Public Health, Lanzhou University, Lanzhou, China; ^2^ College of Basic Medical Sciences, Hubei University of Medicine, Shiyan, China; ^3^ Department of General Surgery, Taihe Hospital, Hubei University of Medicine, Shiyan, China

**Keywords:** irradiation resistance, DNA damage response, PXR, ATF3, MDM2, stabilization, ubiquitination

## Abstract

Low response rate to radiotherapy remains a problem for liver and colorectal cancer patients due to inappropriate DNA damage response in tumors. Here, we report that pregnane X receptor (PXR) contributes to irradiation (IR) resistance by promoting activating transcription factor 3 (ATF3)-mediated ataxia-telangiectasia-mutated protein (ATM) activation. PXR stabilized ATF3 protein by blocking its ubiquitination. PXR–ATF3 interaction is required for regulating ATF3, as one mutant of lysine (K) 42R of ATF3 lost binding with PXR and abolished PXR-reduced ubiquitination of ATF3. On the other hand, threonine (T) 432A of PXR lost binding with ATF3 and further compromised ATM activation. Moreover, the PXR–ATF3 interaction increases ATF3 stabilization through disrupting ATF3–murine double minute 2 (MDM2) interaction and negatively regulating MDM2 protein expression. PXR enhanced MDM2 auto-ubiquitination and shortened its half-life, therefore compromising the MDM2-mediated degradation of ATF3 protein. Structurally, both ATF3 and PXR bind to the RING domain of MDM2, and on the other hand, MDM2 binds with PXR on the DNA-binding domain (DBD), which contains zinc finger sequence. Zinc finger sequence is well known for nuclear receptor peroxisome proliferator-activated receptor-γ (PPARγ) playing E3 ligase activity to degrade nuclear factor κB (NFκB)/p65. However, whether zinc-RING sequence grants E3 ligase activity to PXR remains elusive. Taken together, these results provide a novel mechanism that PXR contributes to IR resistance by promoting ATF3-mediated ATM activation through stabilization of ATF3. Our result suggests that targeting PXR may sensitize liver and colon cancer cells to IR therapy.

## Introduction

Adjuvant or neoadjuvant radiotherapy is applied to treat approximately 50% of all cancers. The rate of pathological complete responses to radiotherapy remains very low (1). Only 12.2% of patients with rectal cancer achieve pathological complete responses after neoadjuvant chemoradiation therapy (2). Resistance to irradiation (IR) therapy occurs in 70%–96% of patients with gastrointestinal cancer, which has become a pressing issue to be solved in order to achieve high rates of pathological complete responses of cancer therapy. Unfortunately, so far, there is no agent that can function as a radiosensitizer to be used in gastrointestinal cancer therapy in the clinic (1).

Cancer cells have been found to employ several mechanisms related to the tumor and the surrounding microenvironment to resist IR damage. Such mechanisms include altered cell cycles, evolving a hypoxia microenvironment, survival from oxidative stress, evading apoptosis, altered DNA damage response (DDR) and enhanced DNA repair, remodeling of cellular energetic system, and acquisition of radioresistant capability (1). Among these mechanisms, DDR is an initiator step to resist radiotherapy for cancer cells and the first step to recover from radioactive damage for healthy cells.

The DDR is a highly organized and coordinated process in eukaryotes to survive DNA damage. This process starts from sensing the aberrant DNA structures induced by genotoxic chemicals and IR, then the signals are transduced and executed to promote survival of organisms. Ataxia-telangiectasia-mutated protein (ATM) is one of the master transducers of DNA damage signal to orchestrate a large network of cellular processes to maintain genomic integrity upon activation. ATM is a kinase that phosphorylates itself and downstream effectors, such as p53 and checkpoint kinase 2 (Chk2), thus arresting cell cycle, allowing for DNA repair or apoptosis. The ATM-Chk2-cell division cycle 25A (Cdc25A)-cyclin-dependent kinase 2 (Cdk2) pathway acts as a genomic integrity checkpoint and guards against radioresistant DNA synthesis (3). ATM phosphorylates and stabilizes zinc (Zn) finger E-box binding homeobox 1(ZEB1) in response to DNA damage, subsequently, ZEB1 interacts with USP7 and enhances its ability to deubiquitylate and stabilize CHK1, thereby promoting CHK1-mediated homologous recombination-dependent DNA repair and resistance to IR in breast cancer cells (4). ATM has become a promising target to develop sensitizers for cancer radiotherapy and chemotherapy.

Activating transcription factor 3 (ATF3) belongs to ATF/cAMP-responsive element-binding protein (CREB) family (5). ATF3 mainly functions as a tumor suppressor, and its expression is induced under stress conditions (6). Previously, researchers reported that ATF3 stabilizes “genome guardian” p53 by blocking murine double minute 2 (MDM2)-mediated ubiquitination, and a cancer-derived ATF3 mutant (R88G) devoid of ubiquitination failed to prevent p53 from MDM2-mediated degradation and thus was unable to activate DDR (7, 8). Additionally, ATF3 is involved in DDR by regulating ATM activation (9, 10). ATF3 promotes acetylation and activation of ATM through recruiting USP7 to Tat-interactive protein 60 (Tip60) that is a MYST histone acetyltransferase (HAT), thus stabilizing Tip60 and facilitating acetylation and phosphorylation of ATM (10). It has been shown that ATF3 promotes resistance of breast cancer to chemotherapy (11, 12) and radiotherapy (13).

MDM2 is an E3 ubiquitin ligase that carries out the final step in the ubiquitination cascade, catalyzing transfer of ubiquitin from an E2 enzyme to form a covalent bond with a substrate lysine (14–16). ATF3 is one of the substrates of MDM2 and degraded through MDM2-mediated ubiquitin–proteasome pathway (17).

Pregnane X receptor (PXR, NR1I2, SXR) belongs to the nuclear receptor superfamily and is mainly expressed in normal liver, small intestine, colon, duodenum, and gall bladder tissues (18). PXR is involved in the metabolism of a broad spectrum of endogenous and xenobiotic compounds, including more than 50% of clinical drugs through coordinately regulating the expression of phase I and phase II drug-metabolizing enzymes (19–21). In addition, PXR is also involved in carcinogenesis, glucose metabolism, and inflammation response (19). Interaction between PXR and PRMT1 promotes translocation of PRMT1 from nuclei to cytoplasm, and interaction between PXR and YAP mediates liver size and regeneration (22, 23). Our previous studies have revealed that PXR plays an important role in protecting hepatocellular carcinoma HepG2 cells against DNA damage induced by benzo[a]pyrene (BaP), a representative genetic toxicant of polycyclic aromatic hydrocarbons (PAHs), probably through a coordinated regulation of genes involved in BaP metabolism (24, 25). These studies suggest that PXR protects liver cancer cells from DNA damage stress. We hypothesized that PXR may also protect cancer cells from DNA damage induced by IR and confers resistance of these cells to IR.

In this study, we investigated the role and the mechanism of PXR in the protection of liver and colon cancer cells from IR-induced DDR. We have found that PXR confers resistance of liver and colon cancer cells to IR-induced DNA damage stress through stabilization of ATF3. In this pathway, PXR promotes ATM activation through ATF3. PXR stabilizes ATF3 against MDM2-mediated ubiquitination through both disrupting MDM2–ATF3 interaction and negatively regulating MDM2 expression level. Our findings provided a clue to overcome resistance of liver and colon cancers to IR therapy by targeting PXR.

## Materials and Methods

### Plasmids

Flag-ATF3 and GST-ATF3, HA-ubiquitin, pCG-ATF3 were kindly provided by Dr. Chunhong Yan (Augusta University) and Dr. Tsonwin Hai (Ohio State University). GST-PXR, GST-PXR (1-107), and GST-PXR (107-434) were kindly presented by Dr. Yanan Tian (Texas A&M University). pCDNA3-MDM2 (Plasmid #16233) and Myc3-HDM2 (Plasmid #20935) were purchased from Addgene (Watertown, MA, USA). The primers used to develop GST-ATF3 truncated protein upon request. PXR protein was purchased from ProteinOne (Rockville, MD, USA). All site-directed mutations of PXR or ATF3 constructs were generated using Quick Change Site-Directed Mutagenesis Kit (Agilent, Santa Clara, CA, USA).

### Cell Culture and Colony Formation Assays

Colon cancer LS180 cells and liver cancer HepG2-C3A cells were obtained from ATCC and cultured in EMEM supplemented with 10% FBS, 100 units/ml penicillin, and 100 µg/ml streptomycin. Cells were verified to be mycoplasma-free by using Mycoplasma Detection Kit (R&D Systems). To generate PXR-knockout (KO) cell line, PXR CRISPR/Cas9 KO plasmid (sc-400824-KO-2) and PXR HDR plasmid (sc-400824-HDR-2) were co-transfected using Lipofectamine 2000 (Invitrogen) according to the manufacturer’s instruction. Here, 2 μg/ml puromycin was used to screen the PXR-KO single clones. For colony formation assays, 1,000 cells were seeded in 6-well plates and were exposed to 0, 10, and 30 Gy of IR, and surviving colonies were fixed with 10% formalin and stained with 0.1% crystal violet 8 days later. Stained colonies were dissolved with buffer containing SDS, and absorbance was recorded at 490 nm using a microplate reader.

### Neutral Comet Assay

Radiated cells were trypsinized, centrifuged for 2 min at 300×g, and resuspended in PBS at 2 × 10^4^/ml. Cell suspensions were then mixed with 1% of low-melting point agarose at 1:3 (V/V) and layered on a microscope slide precoated with 1% agarose gel. Cells in gels were lysed with the neutral lysis buffer (2 M NaCl, 30 mM EDTA, 10 mM Tris-HCl, pH 8.5, 1% N-laurylsarcosine, 1% Triton X-100) at 4°C for 1 h. After rinsing 3 times with the TBE buffer (90 mM Tris, 90 mM boric acid, 2 mM EDTA, pH 8.5) at room temperature for 30 min, slides were submerged in TBE for electrophoresis at 20 V for 25 min. Slides were then rinsed, neutralized in distilled water, dipped in ethanol, and dried. Slides were stained using 2.5 µg/ml of ethidium bromide in distilled water for 20 min and observed under a fluorescence microscope. At least 100 comet images from each slide were analyzed using the CASP software (http://casplab.com/).

### Western Blotting

Cells were lysed in modified radioimmunoprecipitation assay (RIPA) containing 50 mm Tris-HCl (pH 7.4), 150 mm NaCl, 1% Nonidet P-40, 0.1% SDS, 0.5% sodium deoxycholate, 1 mm EDTA, and proteinase inhibitor mixtures. The proteins were resolved in 10%–12% SDS-PAGE gels and detected using the desired antibodies. The antibodies and the dilution ratios are as follows: ATF3 (sc-44C3a, 1:1000), PXR (sc-48340, 1:1000), and HA antibody (sc-805, 1:200) were purchased from Santa Cruz Biotechnology, Inc. (Dallas, TX, USA). MDM2 (4B11, 1:500) was purchased from EMD Millipore (Billerica, MA, USA); anti-mouse IgG VeriBlot for IP secondary antibody (HRP) (ab131368) and anti-Phosphor-(Ser/Thr) Phe antibody (ab17464, 1:1000) were purchased from Abcam (Cambridge, MA, USA). FLAG (F3165, 1:4000) and actin (A5441, 1:5000) were purchased from MilliporeSigma (St. Louis, MO, USA). The p-Ser1981 ATM (#5883, 1:1,000) and p-H2AX (#2557, 1:1000) were purchased from Cell Signaling (Danvers, MA, USA).

### Quantitative RT-PCR

Total RNA was extracted from cells using TRIzol (Invitrogen, Carlsbad, CA, USA), reverse-transcribed using the SuperScript^®^ VILO™ cDNA Synthesis Kit (Thermo Fisher Scientific Inc. NYSE: TMO), and subjected to real-time PCR assays using SYBR Green reagents (Qiagen, Venlo, Netherlands). The sequences of the primers were as follows: GAPDH, 5’-AACGGATTTGGTCGTATTGGG-3’ and 5’-CCTGGAAGATGGTGATGGGATT-3’; ATF3, 5’-GTGCCGAAACAAGAAGAAGG-3’ and 5’-TCTGAGCCTTCAGTTCAGCA-3’; PXR, 5’-GGCCACTGGCTATCACTTCAA-3’ and 5’-TTCATGGCCCTCCTGAAAA-3’.

### SiRNA Knockdown

The PXR and MDM2 siRNA SMARTpool were purchased from Dharmacon (Thermo Fisher Scientific Inc. NYSE: TMO). Double knockdown of PXR and MDM2 or ATF3 expression was carried out using RNAiMAX (Invitrogen, Carlsbad, CA, USA) following the manufacturer’s protocols. Briefly, HepG2-C3A and LS180 cells were transfected with 100 pM SiPXR, Si MDM2, SiATF3, or SiLuc as control for 48 h and aliquoted for further run of knockdown. After 72 h, cells were harvested and subjected to Western blotting assays.

### *In Vitro* GST-Pulldown Assays

In this study, 1 μg GST or GST fusion proteins immobilized on glutathione-agarose from MilliporeSigma (St. Louis, MO, USA) were incubated with *in vitro*-translated proteins (5 μl) (TnT^®^ Quick Coupled Transcription/Translation Systems, L1170, Promega, Madison, WI, USA) or recombinant proteins (50–100 ng) (ProteinOne, Rockville, MD, USA) in a buffer containing 20 mM Tris-HCl, pH 8.0, 100 mM NaCl, 2 mM EDTA, 2 mM DTT, 5% glycerol, and 0.4% NP-40 at 4°C overnight, followed by extensive washes with a similar buffer containing 150 mM NaCl. Bound proteins in the beads were eluted by boiling in the SDS-PAGE loading buffer and detected by Western blotting or fluorography using a desired antibody.

### *In Vivo* Co-Immunoprecipitation Assays

Cell lysates (1–2 mg) were incubated with 20 μl protein A/protein G agarose beads (Cat #: IP05, Millipore) in RIPA buffer (50 mM Tris-HCl, pH 7.4, 100 mM NaCl, 1% NP-40, 0.1% SDS, 0.5% sodium deoxycholate, and 1 mM EDTA) at 4°C overnight for precleaning. After centrifugation, the supernatants were transferred to incubate with another 20 μl of protein A/protein G agarose beads (Millipore) together with 1 μg corresponding antibody in RIPA buffer at 4°C for 5 h. After extensive washes with RIPA buffer containing 150 mM NaCl, bound proteins in the beads were eluted by boiling in the SDS-PAGE loading buffer and detected by Western blotting or fluorography using a desired antibody.

### *In Vivo* Ubiquitination Assays and *In Vitro* Ubiquitination Assays

The *in vivo* ubiquitination assay was performed according to the published method (10). Briefly, H1299 cells were transfected with FLAG-ATF3 and HA-ubiquitin with or without MDM2 or PXR and treated with 5 μM of MG132 overnight and then lysed in the FLAG lysis buffer (50 mM Tris-HCl, pH 7.9, 137 mM NaCl, 10 mM NaF, 1 mM EDTA, 1% Triton X-100, 0.2% sarkosyl, and 10% glycerol). Cell lysates (1–2 mg) were incubated with 20 μl of anti-FLAG M2 affinity gel (Sigma) at 4°C overnight. After extensive washes, agarose gels were loaded on spin columns (Affymetrix), and bound FLAG-ATF3 was eluted with 20 μl of FLAG peptide at a final concentration of 0.1 mg/ml. ATF3 ubiquitination was determined by Western blotting using the hemagglutinin (HA) antibody.

The *in vitro* ubiquitination assay was performed using a human MDM2/HDM2 Ubiquitin Ligase Kit (K-200B) by following the manufacturer’s instructions with modifications. Briefly, *in vitro* translated ATF3 (TnT^®^ Quick Coupled Transcription/Translation Systems, L1170) was incubated with E1, E2, and GST-MDM2, ubiquitin with buffer containing 50 mM Tris-HCl, pH 8.0, 50 mM NaCl, 1 mM EDTA, 5% glycerol, 10 mM fresh DTT on ice for 2 h and then add with or without PXR recombinant protein (ProteinOne, Rockville, MD) at 37°C for another 2 h. Polyubiquitinated proteins were detected using ATF3 antibody or FK-1 (ENZO) after resolving in SDS-PAGE.

### Immunofluorescence Staining

Tissue array that incorporated 50 pairs of human colon adenocarcinoma tissue and adjacent non-tumor tissue sample (D100Co01) from Bioaitech (China) was used to stain the protein expression of PXR (sc-48340, 1: 100) and MDM2 (4B11, 1: 100). Briefly, the tissue array was permeabilized with 0.1% Triton X-100 in PBS solution, and then the array was incubated with the indicated antibody overnight at 4°C, followed by incubation with immunofluorescence second antibody (A32728 and A32742, respectively, Invitrogen) at room temperature for 1 h. The array was washed three times with PBS and sealed with mounting media containing DAPI (Vector Labs, Burlingame, CA, USA; H-2000). The photographs were scanned with 3DHISTECH (PANNORAMIC DESK/MIDI/250/1000, Hungary) and analyzed by ImageJ software.

### Statistical Analysis

Data were analyzed using Prism Software 5.0 (GraphPad Software, Inc.). The statistical significance (p < 0.05) was evaluated by Student’s t test and one-way ANOVA.

## Results

### PXR Physically Interacted With ATF3

**Figure 1:**
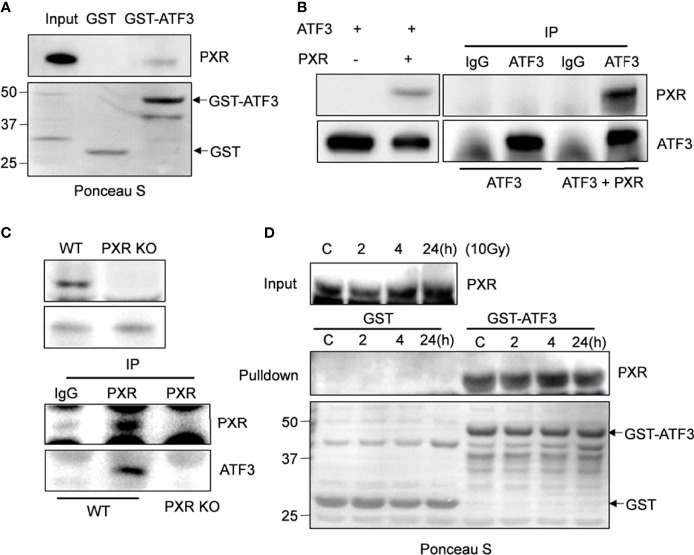
PXR interacts with ATF3. **(A)** PXR interacted with GST-ATF3. 1 μg GST or GSTATF3 protein was immobilized onto 20 μl glutathione agarose beads, and then incubated with 5 μl *in vitro* translated PXR. GST-pulldown assay was performed. **(B)** Interaction between PXR and ATF3 revealed by reciprocal co-IP experiment. H1299 cells in 60 mm3 dish were co-transfected with 3 μg FLAG-PXR, and/or pCG-ATF3. The immunoprecipitation assay was performed using 1μg IgG antibody or ATF3 antibody. **(C)** Interaction between PXR and ATF3 confirmed by endogenous co-IP assay. 3 mg wild-type and PXR-knockout (KO) LS180 cells were harvested, and PXR antibody (sc-48403) was used for immunoprecipitation, HRP-labeled secondary antibody (ab131368) was used to develop signal. **(D)** Irradiation does not alter the binding of PXR to ATF3. LS180 cells were treated with 10 Gy of IR and harvested at different time points. 5 μl *in vitro* translated PXR was incubated with 1 μg immobilized GSTATF3 or GST as indicated for pulldown assays.

Given that ATF3 is quite critical in regulating Tip60 and p53-mediated DDR (7, 10), we want to explore whether PXR binds to ATF3. GST-pulldown assays showed that immobilized GST-ATF3 directly pulled down PXR protein *in vitro.* This was specific since GST alone did not pull down PXR (**Figure 1A**). In order to investigate whether the interaction between PXR and ATF3 occurs endogenously, we performed *in vivo* reciprocal co-immunoprecipitation (co-IP) assays using H1299 cells transiently co-transfected with PXR and/or ATF3 plasmid. The results showed that ATF3 antibody but not IgG precipitated PXR in H1299 cells transfected with both ATF3 and PXR (**Figure 1B**). Since PXR protein was only expressed in the liver cancer HepG2-C3A cells and colon adenocarcinoma LS180 cells (**Supplementary Figure S1A**), we then generated PXR KO of HepG2-C3A and LS180 cells. We found that PXR antibody precipitated ATF3 in wild-type LS180 cells but not in PXR-KO LS180 cells **(** **Figure 1C**). These results suggested that PXR physically interacted with ATF3. Because ATF3 expression alters largely under DNA damage treatments (7), we also determined whether the DNA damage condition affects the interaction between ATF3 and PXR. We then subjected LS180 cells to γ-IR and incubated cell lysates with GST-ATF3 and carried out the endogenous GST-pulldown assay. The results showed that PXR also interacted with ATF3 upon IR (**Figure 1D**, **Supplementary Figure S1B**); furthermore, like the ATF3–Tip60 interaction (7), the binding between PXR and ATF3 was altered neither by time extension after IR treatment nor by DNA damage treatment, such as IR, doxorubicin (DOX), or camptothecin (CPT) (**Figure 1D** and **Supplementary Figure S1B**).

### PXR Increases ATF3 Stability by Blocking Ubiquitination

**Figure 2:**
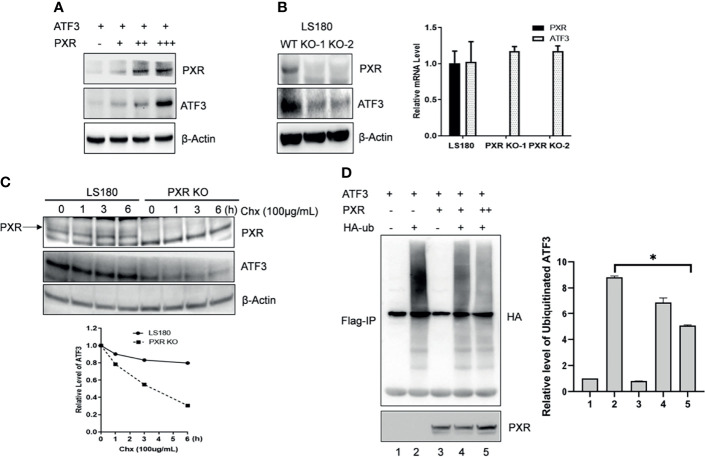
PXR increased ATF3 stability by blocking its ubiquitination. **(A)** Co-transfection of PXR increased the ATF3 protein levels. H1299 cells were transfected with 0.2 μg FLAGATF3 and 0, 0.4, 0.8, or 3.2 μg PXR plasmids as indicated for 2 days. The protein levels were determined using Western blotting. **(B)** PXR regulates ATF3 in protein level. Two LS180 PXR KO clones (KO-1 and KO-2) and the wild-type cells were subjected for Western blotting and quantitative RT-PCR. **(C)** PXR stabilizes ATF3 protein. LS180 wild-type or PXR KO cells were treated with 100 μg/ml of cycloheximide (Chx), and lysed for Western blotting as indicated. Relative ATF3 levels were quantitated by densitometry and presented in the lower plot. **(D)** PXR decreased the ATF3 ubiquitination level. Lysates from H1299 cells transfected with 1μg FLAG-ATF3, 2μg HA-ubiquitin, and/or PXR plasmids (lane 3 and lane 4, 2μg; lane 5, 4ug) were subjected to immunoprecipitation assays using the FLAG agarose beads (Sigma) followed by SDS-PAGE. Ubiquitinated ATF3 were detected by HA antibody. *p < 0.05.

ATF3 protein has a very short half-life, and its protein stability can be altered (17). We then want to know whether PXR can affect the protein stability of ATF3. Results of co-expression with a fixed amount of ATF3 and different doses of PXR expression plasmids showed that the ATF3 protein levels were increased in association with increments of the doses of PXR plasmid (**Figure 2A**). KO of PXR decreased ATF3 expression (**Figure 2B**). The regulation of ATF3 by PXR does not likely occur in the transcriptional level, as there were no significant differences in the ATF3 mRNA levels between the wild-type and the screened PXR-KO clones (**Figure 2B**). Furthermore, this effect is not clone specific, as we transfected HepG2-C3A and LS180 wild-type cells with siRNAs targeting PXR, and the results confirmed that knockdown of PXR resulted in downregulation of ATF3 (**Supplementary Figure S2B**). PXR indeed extended the half-life of endogenous ATF3 protein (**Figure 2C**). These results have suggested that PXR stabilizes ATF3 at the protein level. It has been shown that ATF3 is susceptible to undergo ubiquitination, then proteolytic degradation (17, 26, 27); we next investigate whether ubiquitination of ATF3 is regulated by PXR. Since ATF3 can work as a ubiquitin trapper and FLAG antibody can precipitate ubiquitinated ATF3 (8), we transfected FLAG-ATF3, HA-ubiquitin, with or without PXR, and found that PXR indeed decreased ubiquitination of ATF3 in a dose-dependent manner (**Figure 2D**, lane 5 vs. lane 4). And this effect was proteasome dependent, as ATF3 decreased when ubiquitin was added (**Supplementary Figure S2B**). Taken together, PXR increased ATF3 stability and extended ATF3 protein half-life by blocking its ubiquitination and degradation.

### PXR–ATF3 Interaction Is Required for Regulating ATF3

**Figure 3:**
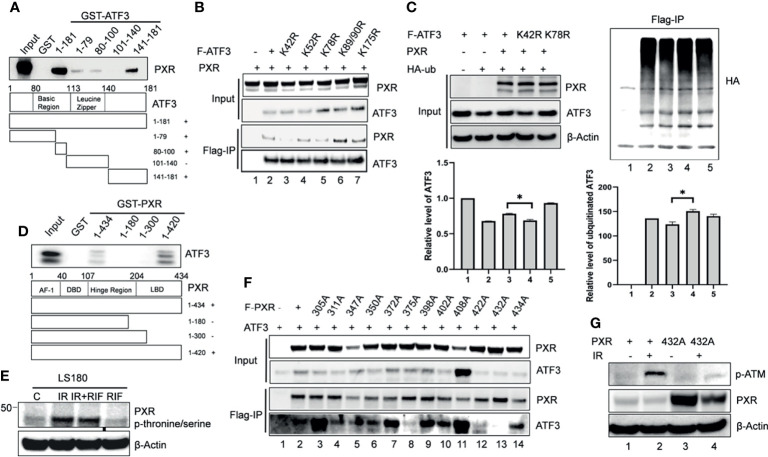
The PXR-ATF3 interaction is required for ATF3 regulation. **(A)** Identification of ATF3 domains for PXR binding. 1 μg ATF3 truncated proteins fused to GST were immobilized onto glutathione agarose beads and incubated with 5 μl *in vitro* translated PXR for GST-pulldown assays. The pulled-down proteins were detected by Western blotting using the PXR antibody. **(B)** The ATF3 K42R mutation significantly reduced the binding between ATF3 and PXR. H1299 cells were transfected with 1.5 μg FLAG-ATF3 wild type or mutants, and 1.5 μg PXR as indicated for 2 days. FLAG-immunoprecipitation (IP) assays were performed using FLAG antibody to precipitate. **(C)** The ATF3 K42 was essential for ubiquitination of ATF3 regulated by PXR. H1299 cells were transfected with 1 μg FLAGATF3 wild type or mutants, and 2μg HA-ubiquitin, and/or 4 μg PXR as indicated for 2 days, and then subjected to FLAG-IP assays using FLAG antibody to precipitate *p < 0.05. **(D)** Mapping of PXR domains/regions for ATF3 binding. 1 μg PXR truncated proteins fused to GST were immobilized onto glutathione agarose beads and incubated with 5 μl *in vitro* translated ATF3 for GST-pulldown assays. The pulled-down proteins were detected by Western blotting using the ATF3 antibody. **(E)** Irradiation-induced significant increases in the phosphorylation of threonine/serine in PXR in colon cancer cells. LS180 cells were treated with 10 μM rifampicin (RIF) for 12 h, then radiated at a dose 10 Gy for 4h, PXR antibody was used to immunoprecipitate, then anti- Phosphor - (Ser/Thr) Phe antibody (ab17464) was used to blot. **(F)** The PXR T432A mutation significantly reduced the binding between ATF3 and PXR as determined by FLAG-IP. H1299 cells were transfected with 1 μg FLAG-PXR wild type or mutants, and 2μg HA-ubiquitin, and/or 1 μg ATF3 as indicated for 2 days, and then subjected to FLAG-IP assays using FLAG antibody to precipitate. **(G)** The PXR T432A mutation abolished ATM phosphorylation and activation induced by IR in colon cancer cells. LS180 PXR knockout cell was transfected with 1.5 ug PXR wildtype, or mutant for 2 days, and then subjected to 10 Gy of γ- irradiation and harvested after 24 h. The protein levels were determined by Western blotting using the indicated antibodies.

We next want to figure out whether interactions between PXR and ATF3 are required for PXR-mediated regulation of ATF3. In order to gain this aim, we built several truncated constructs of ATF3 fused with GST agarose beads, incubated with *in vitro* translated PXR, and performed GST-pulldown assay. We found that full-length ATF3 except for leucine zipper (Zip) domain bound with PXR as a fragment of aa 1-79, aa 80-100, aa 141-181 was required for ATF3 to interact with PXR (**Figure 3A**). We therefore want to know whether there is an ATF3 binding-deficient lysine (K) residue that loses the ability to be ubiquitinated by PXR. Normally, lysine residue on the protein structure can be substrate to attach ubiquitination cascade (14), then we mutated 6 lysine residues on the binding region of ATF3 to arginine (R), performed FLAG-IP to test which mutant will lose the binding with PXR, and further abolished PXR-mediated regulation. Indeed, K42R mutation failed to pull down PXR (**Figure 3B**, lane 3 vs. lane 2). Furthermore, the FLAG-tagged ATF3 K42R mutation abolished PXR-mediated reduction of ubiquitination of ATF3 (**Figure 3C**, lane 4 vs. lane 3). The results suggested that the ATF3 K42 in a fragment of aa 1-79 was essential for PXR-inhibited ubiquitination of ATF3.

On the other hand, GST-pulldown assay revealed that partial ligand-binding domain of PXR (aa 300-434) was required for PXR to interact with ATF3 as a truncated fragment of aa 1-434, aa 1-420 but not aa 1-180, or aa1-300 pulled down ATF3 (**Figure 3D**). PXR protein structure is enriched with serine (S)/threonine (T) amino acid and defined as a phosphoprotein (28). Indeed, we observed that IR induced significant increases in the phosphorylation of S/T in PXR (**Figure 3E**). In order to identify which S/T sites are essential for PXR–ATF3 interaction, we mutated 12 S/T sites to alanine (A), which is located on aa 300-434 of PXR-binding region, and we found that the T432A mutant loses the ability to interact with ATF3 (**Figure 3F**, lane 13 vs. lane 2). In order to confirm that the phosphorylation of T432 is critical for ATF3-mediated ATM activation upon IR, we transfected the wild-type PXR or the PXR T432A mutant plasmid into the PXR-KO LS180 cells and then subjected cells to IR. The results demonstrated that the p-ATM level was significantly decreased in the PXR-KO LS180 cells transfected with the PXR T432A mutant plasmid compared with that of the wild-type PXR transfection (**Figure 3G**, lane 6 vs. lane 4), suggesting that the phosphorylation of PXR T432 was pivotal for ATF3-mediated ATM activation upon IR in colon cancer cells.

### PXR Stabilized ATF3 by Counteracting MDM2-Catalyzed Ubiquitination of ATF3

**Figure 4:**
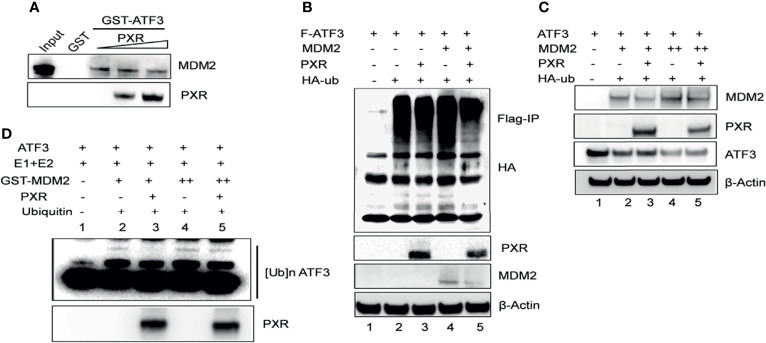
PXR counteracts MDM2-mediated ubiquitination and stabilization of ATF3. **(A)** PXR disrupted ATF3-MDM2 interaction *in vitro*. *In vitro* translated MDM2 and GST-ATF3 were mixed and incubated for 4 h, then 0.5 μg or 1 μg recombinant PXR proteins were added and incubated at 4°C overnight. GST- pulldown assay was performed and the protein levels were determined by Western blotting using indicated antibodies. **(B)** PXR decreased MDM2-catalysed ubiquitination of ATF3. H1299 cells were transfected with 0.5 μg FLAG-ATF3, 2μg MDM2, HA-ubiquitin, and/or 3μg PXR plasmids. After cells were lysed, FLAG-IP assay was performed using FLAG antibody to precipitate and HA antibody to probe. **(C)** PXR counteracted MDM2-induced downregulation of ATF3. H1299 cells were transfected with 0.2μg ATF3, 0.8 μg PXR, and increasing amounts of MDM2 (+, 0.8μg; ++, 1.6μg) plasmids as indicated, and then lysed for Western blotting. **(D)** PXR protected ATF3 against MDM2-mediated ubiquitination *in vitro*. *In vitro* translated ATF3 incubated with E1, E2, and/or GSTMDM2 (E3) for 2h at 4°C, then mixed with or without 1μg recombinant PXR protein at 37°C for 2h. The protein levels were determined by Western blotting using indicated antibodies.

Besides p53, ATF3 is a well-known substrate of E3 ligase MDM2, and MDM2 is responsible for the ubiquitination and degradation of ATF3 (17). This knowledge leads us to investigate whether PXR disrupts ATF3–MDM2 interaction, thus abrogating MDM2-mediated ubiquitination and stabilizing ATF3. To gain this aim, we incubated *in vitro* translated MDM2 with glutathione agarose beads conjugated with ATF3 overnight, added recombinant PXR to the system, and performed GST-pulldown assay. The results showed that ATF3 pulled down MDM2 as expected, and the amount of MDM2 bound with ATF3 was decreased by addition of recombinant PXR in a dose-dependent manner, suggesting that PXR indeed disrupted ATF3–MDM2 interaction *in vitro* (**Figure 4A**). Next, we want to clarify whether PXR increases ATF3 stability by blocking MDM2-catalyzed ATF3 ubiquitination. By applying FLAG-IP, we found that the ubiquitination levels of ATF3 were increased by MDM2, whereas PXR abolished MDM2-mediated ubiquitination (**Figure 4B**, lanes 5 vs. lane 4). By applying Western blotting, we found that the ATF3 protein levels were decreased by MDM2, while these were increased by addition of PXR (**Figure 4C**, lane 3 vs. lane 2, lane 5 vs. lane 4). Furthermore, we also performed *in vitro* ubiquitination assay, and the results demonstrated that the ATF3 ubiquitination levels were enhanced by MDM2 in a dose-dependent manner (**Figure 4D**, lane 2 and lane 4) as expected. More importantly, addition of PXR reduced the MDM2-mediated ATF3 ubiquitination (**Figure 4D**, lane 3 vs. lane 2 and lane 5 vs. lane 4). However, PXR is not an E3 ligase for ATF3, and it has no capability to induce ubiquitination of ATF3 (**Supplementary Figure S4E**, lane 6 vs. lane 3). Collectively, our results indicated that PXR suppressed MDM2-catalyzed ubiquitination of ATF3 *in vivo* and *in vitro*.

### PXR Interacts With MDM2 and Negatively Regulates MDM2 Protein Expression

**Figure 5:**
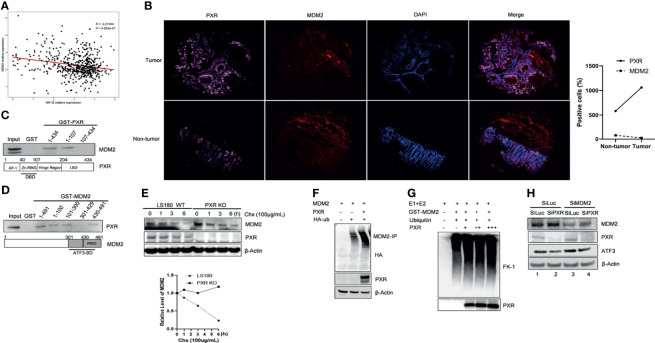
PXR interacts with MDM2 and negatively regulate expression of MDM2. **(A)** mRNA expression of PXR negatively correlated with mRNA expression of MDM2. 151 colorectal samples from TCGA database were collected and analyzed the correlations between mRNA level of PXR and that of MDM2. **(B)** PXR, MDM2 immunofluorescence staining in matched samples of human colon adenocarcinoma tissue and adjacent non-tumor tissue. The right panel shows the statistics of positive staining cells. n = 50 for each group. **(C)** The DBD contains zinc-finger region of PXR physically interacted with MDM2. Truncated PXR were immobilized with GST and incubated with *in vitro* translated MDM2 and subjected with GST-pulldown to map the region of PXR binding with MDM2. **(D)** PXR physically interacted with MDM2 on the RING domain. Truncated MDM2 were immobilized with GST and incubated with *in vitro* translated PXR and subjected with GST-pulldown to map the region of MDM2 binding with PXR. **(E)** PXR reduced MDM2 protein half-life in colon cancer cells. LS180 wild-type and PXR knockout cells were treated with 100 μg/mL cycloheximide (CHX), harvested at different time point, and then Western blotting was performed to detect MDM2 half-life alteration. Relative MDM2 levels were quantitated by densitometry and presented in the right plot. **(F)** Ubiquitination of MDM2 was enhanced by addition of PXR. H1299 cells were transfected with 0.5 μg HDM2, 1 μg HA-ubiquitin, and/or 2 ug PXR plasmids, then MDM2-immunoprecipitation was performed with HA antibody to probe. **(G)** PXR increased polyubiquitination of MDM2 in a dose-dependent manner. E1, E2, and GST-MDM2 (E3) were incubated with ubiquitin for 3 h at 4°C, and 0.5 μg, 1 μg, 3 μg recombinant PXR was then added as indicated and incubated for another 3 h. The protein levels were determined by Western blotting. FK-1 antibody was used to detect polyubiquitination. **(H)** MDM2 inhibition by siRNA abolished knockdown of PXR mediated downregulation of ATF3 expression. LS180 cells were transfected with 20 nM SiLuc or SiMDM2 for 24 h and then transfected with 20 nM SiLuc or SiPXR for 48 h. Then cells were collected and lysed, and the PXR, MDM2 and ATF3 protein levels were determined using Western blotting.

Interestingly, we noticed a diminished band of MDM2 when we co-transfected MDM2 with PXR plasmid together (**Figure 4B**, lane 5 vs. lane 4), which indicated that PXR might affect MDM2 expression level. To further understand this regulation, we analyzed 151 samples from The Cancer Genome Atlas rectum adenocarcinoma (TCGA-READ) database (portal.gdc.cancer.gov/) and found that the mRNA expression level of PXR negatively correlated with mRNA expression of MDM2 (p < 0.05) (**Figure 5A**). Immunofluorescence staining has revealed that PXR protein level is ubiquitously higher in human colon adenocarcinoma tissues than that in adjacent non-tumor tissues, whereas MDM2 protein expression has the opposite trend with PXR in tumor tissue vs. that in adjacent non-tumor tissues (**Figure 5B**). Indeed, PXR has physical binding with MDM2. By performing GST-pulldown assay, we found that MDM2 bound with PXR in the DNA-binding domain (DBD) (aa 1-107) (**Figure 5C** and **Supplementary Figure S4A**). Conversely, PXR binds with MDM2 on the RING domain (aa 431-491) (**Figure 5D** and **Supplementary Figure S4B**). The RING domain of MDM2 is essential for its E3 ligase activity. Coincidently, ATF3 also binds with MDM2 on this RING domain. The structure binding analysis provides the explanation that PXR structurally can interrupt the interaction between ATF3 and MDM2. In order to further address PXR-inhibited MDM2 expression, we detected the half-life of MDM2 and ubiquitination of MDM2. Strikingly, the result showed that PXR reduced the MDM2 half-life in colon cancer cells (**Figure 5E**), and PXR increased endogenous ubiquitination of MDM2 (**Figure 5F**). MDM2 is an oncogene, and its auto-ubiquitination enhances its substrate ubiquitin ligase activity (29). In order to further confirm that PXR increases auto-ubiquitination of MDM2, we performed *in vitro* ubiquitination assays, incubated E1 and E2, and purified GST-MDM2 and recombinant PXR *in vitro* together. FK-1 antibody was used to detect polyubiquitination (7). The results showed that PXR indeed increased auto-ubiquitination of MDM2 in a dose-dependent manner (**Figure 5G**). In order to further confirm that PXR is an upstream regulator for MDM2, we transfected siRNA targeting PXR, and the results showed that PXR knockdown resulted in downregulation of ATF3 and upregulation of MDM2 (**Figure 5H**, lane 2 vs. lane 1, **Supplementary Figure S4C**). More importantly, it seems that PXR expression level is not altered by MDM2 (**Figure 5G**, lane 3 vs. lane 1), whereas MDM2 expression is dependent on PXR regulation (**Figure 5H**, lane 4 vs. lane 2). As a consequence, like ATF3, tumor suppressor p53, both of which are substrates of MDM2, also can be upregulated by PXR (**Supplementary Figure S4D**).

### PXR Confers Irradiation-Induced DNA Damage Resistance Through Regulating ATF3

**Figure 6:**
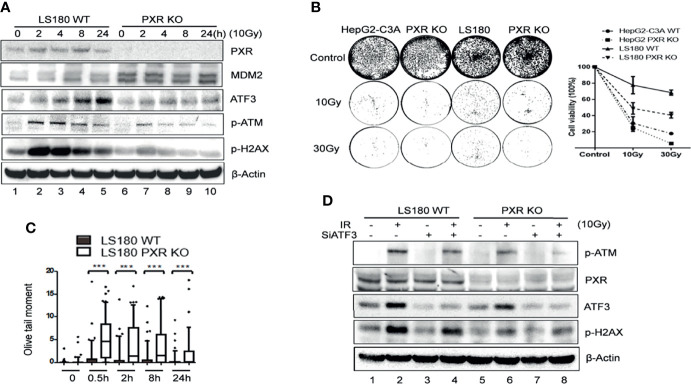
Knockdown of PXR expression impaired ATM activation by IR. **(A)** IR-induced ATM activation and phosphorylation of downstream substrates were repressed in PXR knockout LS180 cells. Wildtype LS180 cells or PXR KO cells were irradiated (10 Gy), and then subjected to Western blotting using the indicated antibodies. **(B)** PXR promoted cell viability of LS180 cells against irradiation. Cells were irradiated and subjected to colony formation assay in 60-mm dishes in triplicate. The colonies in the well were counted by Image J and quantified in the right panel. DNA lesions were accumulated in PXR knockout LS180 cells after radiation. **(C)** PXR promoted DNA damage repair. Cells were radiated at a dose 10 Gy, and then used for neutral comet assays at the indicated time after radiation. ***p < 0.001, compared with the control, MannWhitney U-test. **(D)** PXR promotes ATM signaling through regulating ATF3. LS180 wildtype or PXR KO cells were transfected with 100 pmol ATF3 siRNA (siATF3) or control siRNA (siLuc) for 3 days, then subjected to 10 Gy of γ-radiation. The protein levels of PXR, ATF3, p-ATM, and p-H2AX were determined using Western blotting. Beta-actin was used as an internal control.

Previously, we proved that ATF3 promoted ATM activation upon IR treatment *via* USP14-Tip60 axis. Consistent with these results, IR-induced phosphorylation of ATM was dramatically suppressed in the PXR-KO cells where the ATF3 expression level was downregulated (**Figure 6A**, lanes 6–10 vs. lanes 1–5). Similarly, IR-induced phosphorylation of ATM substrates including H2AX was also repressed in the PXR-KO cells (**Figure 6A**). The suppression of ATM activation by PXR KO was not limited to LS180 cells, as similar observations were obtained in HepG2-C3A PXR-KO cells (**Supplementary Figure S5A**). Meanwhile, upregulated MDM2 followed PXR KO (**Figure 6A**). In line with this result, there are more colonies in the wild-type LS180 and HepG2-C3A cells after IR than those in PXR-KO cells (**Figure 6B**). Besides, the olive tail moments of the wild-type LS180 cells were much shorter than those of the PXR-KO LS180 cells (**Figure 6C**). These results suggested that PXR promoted cell viability and conferred IR resistance through facilitating ATM activation of DNA damage response and repair in liver and colon cancer cells. In order to confirm that PXR promotes ATM signaling through regulating ATF3, we knocked down ATF3 expression by siRNA in both wild-type and PXR-KO LS180 cells and then determined IR-induced ATM activation. Consistent with our previous report, knockdown of ATF3 obviously compromised ATM activation (**Figure 6D** and **Supplementary Figure S5B**, lane 4 vs. lane 2), and KO of PXR indeed downregulated ATF3. Furthermore, KO of PXR induced much more suppression of ATM activation where ATF3 expression was further inhibited (**Figure 6D** and **Supplementary Figure S5B**, lane 8 vs. lane 4). Surprisingly, the basal level of phosphorylated ATM was almost abolished when ATF3 expression was knocked down in PXR-KO HepG2-C3A cells (**Supplementary Figure S5B**).

## Discussion

Resistance to IR therapy is a noxious problem in gastrointestinal cancer adjuvant or neoadjuvant radiotherapy. No radiosensitizer has been approved for use in gastrointestinal cancers (1). The rate of pathological complete responses to radiotherapy remains low (1). In the current study, our data reveal that PXR promotes viability and reduces DNA damage of liver and colon cancer cells after IR, providing a clue to overcoming radioresistance in liver and colon cancers where PXR is expressed by targeting PXR.

In the current study, our data have disclosed that PXR protects liver and colon cancer cells from IR-induced DNA damage through ATF3-mediated ATM activation. This is an extension for the mechanism by which PXR protects liver cancer HepG2 cells from DNA damage induced by genotoxicants such as BaP. PXR reduced BaP-induced DNA adduct formation through a coordinated regulation of genes involved in xenobiotic metabolism (24) and through inhibiting the BaP biotransformation (25). Ionized radiation directly causes lethal double-stranded DNA breaks, in which ATM is the master regulator of DNA damage response and repair (30). It is likely our data revealed a novel role of PXR in protecting cells against double-stranded DNA breaks, in addition to single-stranded DNA breaks and DNA adducts.

It has been shown that ATM activation is regulated by ATF3 in response to DNA damage stress (9, 10). As one of the key regulators of the DDR, ATF3 promotes DDR by facilitating ATM activation through increasing Tip60 HAT activity and determining cell fate by regulating p53 stabilization, thereby allowing appropriate cellular response to DNA damage (7, 10, 31). Despite many events that are regulated by ATF3 have been elucidated, little is known about how ATF3 activity is regulated. In the current study, we identified the nuclear receptor PXR as an ATF3 regulator. PXR interacts with ATF3 and increases ATF3 protein stability, thus facilitating DDR by promoting ATM activation. PXR is a well-known ligand-regulated transcription factor, and our current study shows that ATF3 protein levels are closely associated with PXR protein levels and PXR protein increases ATF3 protein levels but not the mRNA levels, suggesting that PXR regulates ATF3 at the protein levels. Furthermore, PXR increases ATF3 stability by blocking its ubiquitination. PXR increases ATF3 levels by counteracting MDM2-catalyzed ubiquitination of ATF3 *in vitro* and *in vivo*. Since ATF3 is a *bona fide* substrate of E3 ligase MDM2, MDM2 is the only E3 ligase so far known to degrade ATF3 (17), and ubiquitination is a proteasome-mediated pathway for protein degradation (32), it seems that PXR regulated ATF3 through modulating MDM2-mediated ATF3 protein ubiquitination and degradation, which are independent of its transcription activity. This is also a novel mechanism for PXR to function through protein–protein interaction and signaling crosstalk in the regulation of cellular function (19), in addition to being a transcription factor.

Strikingly, our data revealed that by binding with RING domain of MDM2, PXR decreased MDM2 protein expression, enhanced polyubiquitination of MDM2, and reduced MDM2 protein half-life (**Figure 5**). It has been reported that the nuclear receptor peroxisome proliferator-activated receptor-γ (PPARγ) exhibits the RING-finger E3 ligase activity and mediates degradation of MUC1-C oncoprotein (33) and nuclear factor κB (NF-κb)/p65 (34). Structurally, the RING-finger E3 ligase contains the Zn fingers of 40–60 residues that bind two atoms of Zn, which mediates protein–protein interactions (35–37). The basic pattern in Zn finger domain is CX (2) CX (9 to 39) CX (1 to 3) HX (2 to 3) CX (2) CX (4 to 48) CX (2) C. One of the characteristic structures of nuclear receptor superfamily is its conserved DBD, which contains Zn finger domain (36, 37). PPARγ contains two C4-type Zn finger domains in the DBD (38), in which a region between amino acid residues 139 and 198 (between the Znf1 and Znf2) is identified to be homologous to the RING domain, which mediated ubiquitination and degradation of NF-κB/p65 (34). Likewise, The DBD of human PXR also consists of two C4-type Zn finger domains in the DBD, CX2CX13CX2C (Zn finger I) and CX5CX9CX2C (Zn finger II) (39). However, whether the Zn fingers in the DBD of PXR contain the E3 ligase activity remains to be elusive.

MDM2 has several substrates including p53 and USP7 and regulates their protein turnover (7, 40). Accelerated MDM2 auto-degradation induced by DNA damage is required for p53 activation (41). Upon DNA damage, activated p53 and MDM2 form a negative feedback loop for a tight regulation of p53 activity. ATF3 is also a stabilizer for p53 by binding to C-terminal of p53 and blocking MDM2-mediated ubiquitination and degradation in response to DNA damage (7). Predictably, PXR might also stabilize p53 by blocking MDM2-mediated ubiquitination and stabilizing ATF3; indeed, we observed co-expression with PXR increased p53 expression (**Supplementary Figure S4D**). Taken together, PXR promoting ATM activation in response to DNA damage is a synergistic effect of upregulation of ATF3 and p53 and so on.

PXR has been shown to be expressed in a variety of cancers, such as breast, prostate, endometrial, ovarian, colon, liver, and gastric cancers. PXR regulates genes involved not only in drug metabolism (19–21) but also in proliferation, metastasis, apoptosis, anti-apoptosis, inflammation, and oxidative stress in cancers; PXR expression is correlated with drug resistance and prognosis of cancer (19, 42, 43). In the current study, our data showed that PXR promotes viability of liver and colon cancer cells upon IR treatment. We concluded that PXR plays an important role in its contribution of resistance to IR-induced damage in liver and colon cancer cells. Inhibition of PXR may be an important adjuvant therapy to increase radiosensitization in cancers, taking advantage of PXR-ATF3-ATM signaling pathway. Our data provide a rationale for clinical development of PXR antagonists for treating IR resistant to anticancer therapies that depend on promoting DNA damage response and repair (44). PXR antagonists ketoconazole, fucoxanthin (FUC), and SPA70 were reviewed in reference (44). Pharmaceutical inhibition of PXR-ATF3-ATM pathway by PXR antagonists will sensitize cells to DNA damage and dampen the cell survival in a cancer cell that was treated with IR. Therefore, understanding the epigenetic regulation of ATF3 by PXR in tumors, such as deletion, mutation, or posttranslational modifications, may help to determine more effective therapeutic methods for cancer patients. The mechanisms documented here might also be implicated in other cancer types that depend on the PXR signaling pathway.

**Figure 7:**
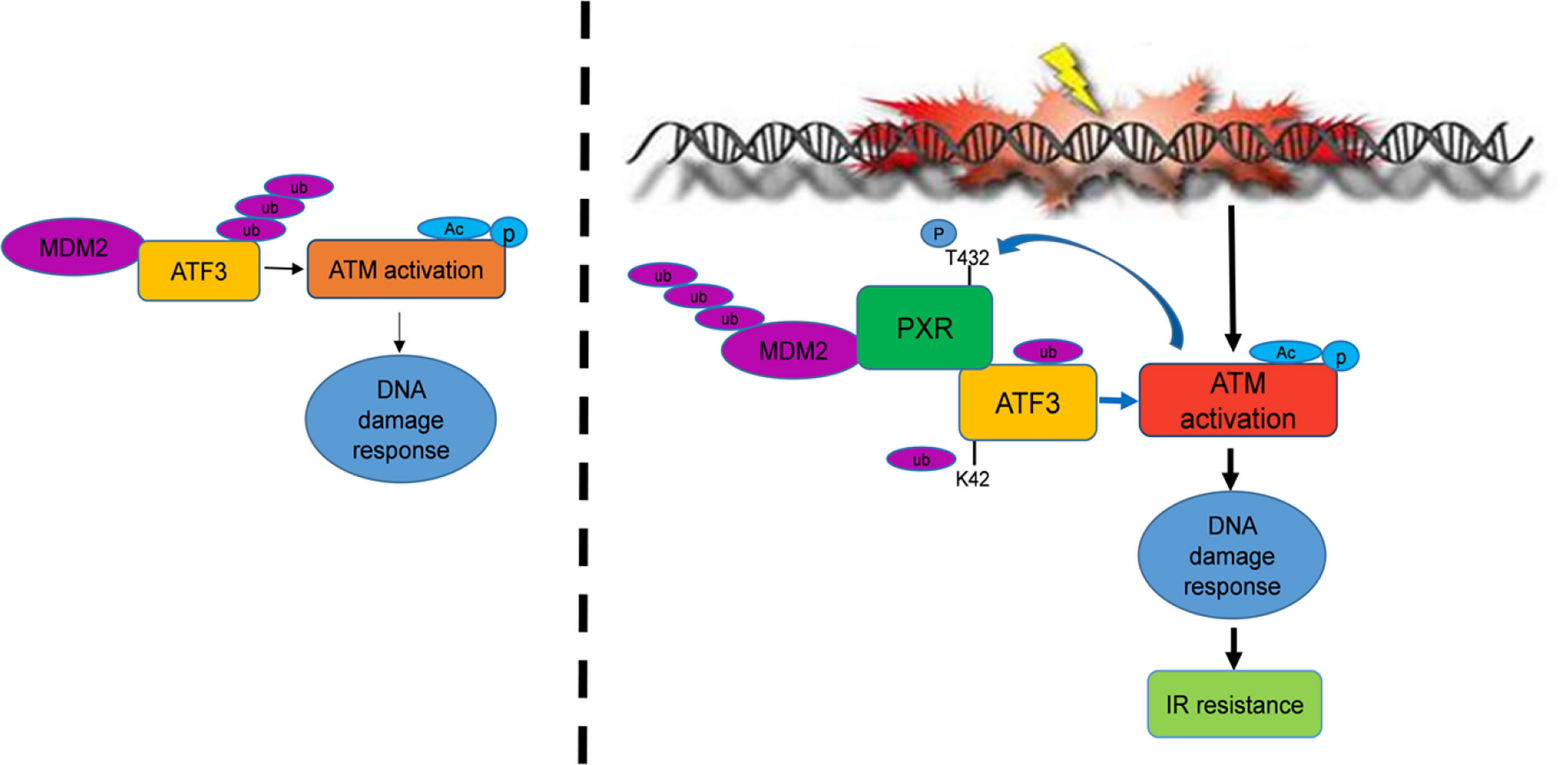
PXR confers to IR resistance by promoting ATF3 stabilization. PXR stabilization of ATF3 protein contains two mechanisms: (1) PXR disrupts ATF3-MDM2 interaction, thus counteracts MDM2-mediated ubiquitination of ATF3. (2) PXR negatively regulates the expression of MDM2 and enhances its auto-ubiquitination and further reduces its half-life. PXR-ATF3 interaction is required for PXR-mediated stabilization of ATF3 as K42R of ATF3 lost binding with PXR and further abrogated PXR-mediated reduction of ATF3 ubiquitination. Furthermore, PXR-ATF3 interaction is also required for ATF3-mediated ATM activation as T432A mutant of PXR lost binding with ATF3, thus compromised ATF3-mediated ATM activation. Taken together, PXR promotes ATM activation and confers liver and colon cancer cell resistance to IR-induced DNA damage.

In summary, in the current study, we find that PXR confers resistance of liver and colon cancer cells to IR-induced DNA damage stress through stabilization of ATF3, thus promoting ATF3-mediated ATM activation. In this pathway, PXR promotes ATM activation through ATF3. PXR stabilizes ATF3 from MDM2-mediated ubiquitination through both disrupting MDM2–ATF3 interaction and negatively regulating MDM2 expression, promoting MDM2 ubiquitination and degradation (**Figure 7**). Collectively, our findings provide a clue to overcoming resistance of liver and colorectal cancer to IR therapy by targeting PXR.

## Data Availability Statement

The original contributions presented in the study are included in the article/**Supplementary Material**. Further inquiries can be directed to the corresponding authors.

## Author Contributions

HC designed and drafted the raw article. HC and XN performed most of the experiments. HC and XG reviewed and edited the article. TW, MS, CZ, and MM performed some of the experiments. All authors contributed to the article and approved the submitted version.

## Funding

This work was supported by the initiative fund for faculty development from Lanzhou University (# 561119203).

## Conflict of Interest

The authors declare that the research was conducted in the absence of any commercial or financial relationships that could be construed as a potential conflict of interest

## Publisher’s Note

All claims expressed in this article are solely those of the authors and do not necessarily represent those of their affiliated organizations, or those of the publisher, the editors and the reviewers. Any product that may be evaluated in this article, or claim that may be made by its manufacturer, is not guaranteed or endorsed by the publisher.
