# Supplementary material for: Nuclear Receptor PXR Confers Irradiation Resistance by Promoting DNA Damage Response Through Stabilization of ATF3
Source: Front Oncol. 2022 Mar 16;12:837980. doi: 10.3389/fonc.2022.837980 (PMC8965888; doi:10.3389/fonc.2022.837980)
Supplement: Supplementary file 1 [file DataSheet_1.docx]

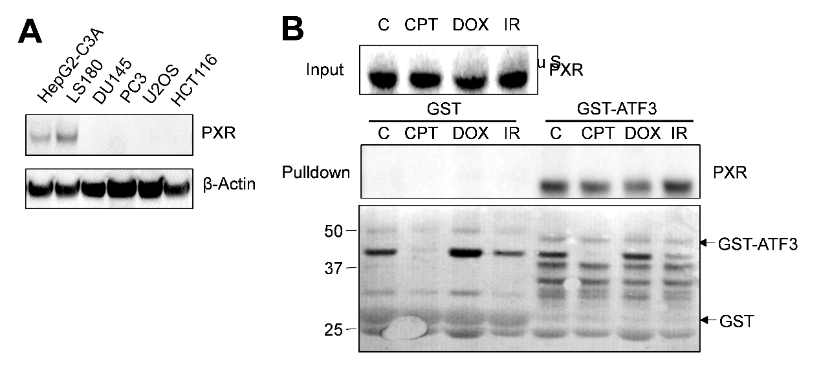


**Supplemental FIGURE 1 |** Different treatment does not alter the binding of PXR to ATF3. **(A)** The PXR protein expression profiles in different cancer cell lines. Cultured cells were collected and lysed with RIPA buffer. The protein levels were determined using Western blotting. **(B)** Indicated treatment does not alter the binding of PXR to ATF3. LS180 cells were treated with 1 μM doxorubicin (Dox), 2 μM camptothecin (CPT), or 10 Gy IR and incubated with 1μg immobilized GST-ATF3 or GST as indicated.


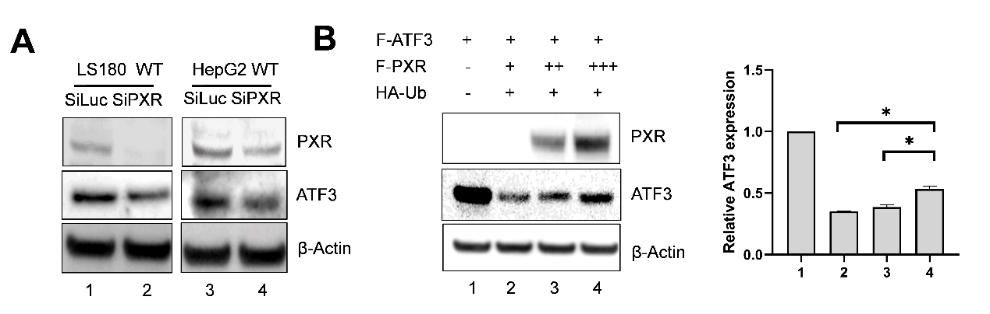


**Supplemental FIGURE 2 |** PXR regulates proteasome-dependent degradation of ATF3. **(A)** Knockdown of PXR by siRNA resulted in decreases in the ATF3 levels in HepG2-C3A and LS180 wild-type cells. **(B)** The regulation of ATF3 turnover by PXR is proteasome dependent. H1299 cells were transfected with 0.8 μg FLAG-ATF3, 1μg HA-ub, and 1.6μg or 3.2 μg PXR plasmids as indicated for 36h, then added 5μΜ MG132 and incubated overnight. The protein levels were determined using Western blotting.


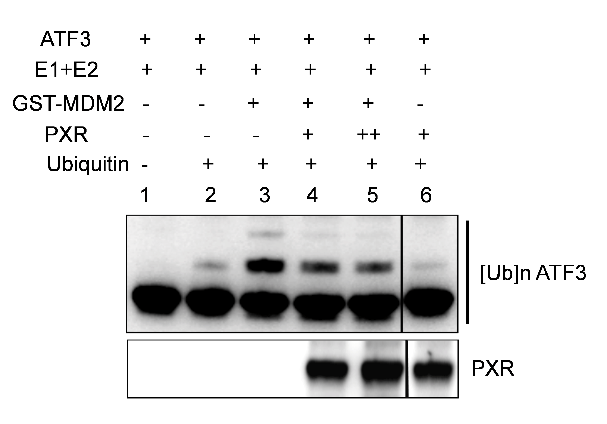


**Supplemental FIGURE 3|**PXR counteracts MDM2-mediated ubiquitination of ATF3 and PXR is not E3 ligase for ATF3. *In vitro* translated ATF3 incubated with E1, E2, and/or GST-MDM2 (E3) for 2h at 4℃, then mixed with 1 μg, or 3 μg of recombinant PXR protein at 37℃ for 2h. The protein levels were determined by Western blotting using indicated antibodies.


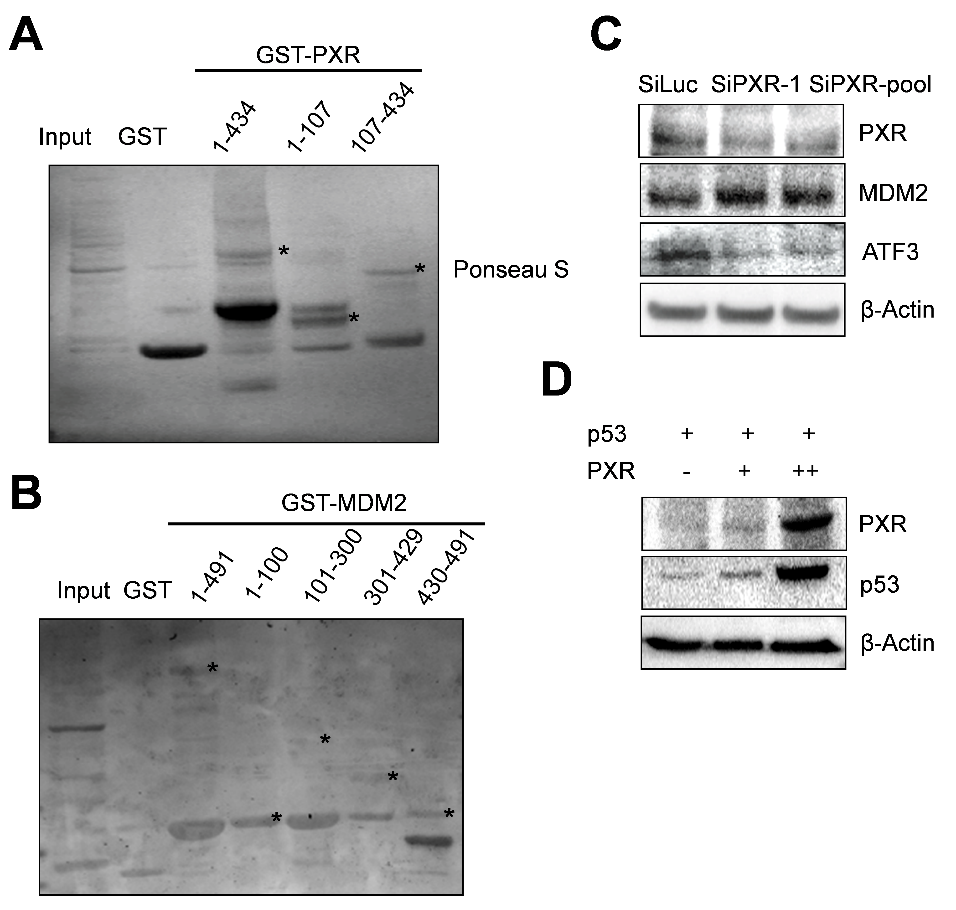


**Supplemental FIGURE 4**| PXR increased ATF3 levels by reducing MDM2 levels in colon cancer cells. **(A)** Ponseau S staining of GST-PXR in FIGURE. 5a. **(B)** Ponseau S staining of GST-MDM2 in FIGURE. 5b. **(C)** PXR increased ATF3 levels by reducing MDM2 levels in colon cancer cells. LS180 cells were transfected with 20 nM SiLuc or SiPXR for 72h. Then cells were collected and lysed, and the PXR, MDM2 and ATF3 protein levels were determined using Western blotting. **(D)** PXR also increased p53 protein expression. Co-expression of PXR increased the p53 expression level. H1299 cells were transfected with 0.5 μg of p53, and/or 0.5 μg, 2 μg of PXR for 2 days, and then subjected to Western blotting.


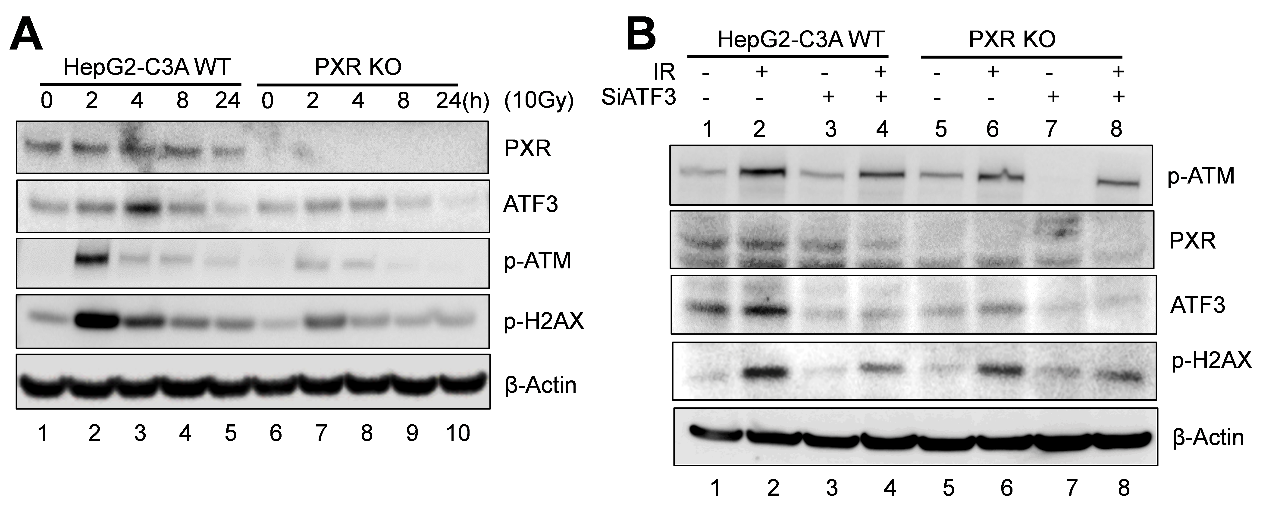


**Supplemental FIGURE 5** | PXR knockout compromised DNA damage response through repressing ATF3 in HepG2-C3A cells. **(A)** Wildtype HepG2-C3A cells or PXR KO cells were irradiated (10 Gy), and then subjected to Western blotting using the indicated antibodies. **(B)** PXR facilitate ATM signaling through regulating ATF3. HepG2-C3A wildtype or PXR KO cells were transfected with 100 pmol ATF3 siRNA (siATF3) or control siRNA (siLuc) for 3 days, then subjected to 10 Gy of γ-radiation. The protein levels of PXR, ATF3, p-ATM, and p-H2AX were determined using Western blotting. Beta-actin was used as an internal control.
